# Knockdown of Mediator Complex Subunit 19 Suppresses the Growth and Invasion of Prostate Cancer Cells

**DOI:** 10.1371/journal.pone.0171134

**Published:** 2017-01-26

**Authors:** Shengqiang Yu, Yanwei Wang, Hejia Yuan, Hongwei Zhao, Wei Lv, Jian Chen, Fengchun Wan, Dongfu Liu, Zhenli Gao, Jitao Wu

**Affiliations:** 1 Department of Urology, the Affiliated Yantai Yuhuangding Hospital of Qingdao University, Yantai, Shandong, China; 2 Central Laboratory, the Affiliated Yantai Yuhuangding Hospital of Qingdao University, Yantai, Shandong, China; 3 Department of Nephrology, the Affiliated Yantai Yuhuangding Hospital of Qingdao University, Yantai, Shandong, China; University of South Alabama Mitchell Cancer Institute, UNITED STATES

## Abstract

Prostate cancer (PCa) is one of the most common cancers in elderly men. Mediator Complex Subunit 19 (Med19) is overexpressed and plays promotional roles in many cancers. However, the roles of Med19 in PCa are still obscure. In this study, by using immunohistochemical staining, we found higher expression level of Med19 in PCa tissues than in adjacent benign prostate tissues. We then knocked down the Med19 expression in PCa cell lines LNCaP and PC3 by using lentivirus siRNA. Cell proliferation, anchor-independent growth, migration, and invasion were suppressed in Med19 knockdown PCa cells. In nude mice xenograft model, we found that Med19 knockdown PCa cells formed smaller tumors with lower proliferation index than did control cells. In the mechanism study, we found that Med19 could regulate genes involved in cell proliferation, cell cycle, and epithelial-mesenchymal transition, including P27, pAKT, pPI3K, IGF1R, E-Cadherin, N-Cadherin, Vimentin, ZEB2, Snail-1 and Snail-2. Targeting Med19 in PCa cells could inhibit the PCa growth and metastasis, and might be a therapeutic option for PCa in the future.

## Introduction

Prostate cancer (PCa) is one of the most common cancers in western world, with a leading cause of mortality and morbidity [1]. Early detection and treatment have improved the survival rate significantly. However, 20~40% of patients undergoing radical prostatectomy and 30~50% of patients undergoing radiotherapy will have biochemical recurrence within 10 years [2]. For advanced PCa, androgen deprivation therapy (ADT) is still the first therapeutic option [3]. Although initially effective at blocking tumor progression, ADT eventually fails by leading PCa to a castration-resistant stage. Many efforts have been made to seek more effective therapeutic methods for PCa [4].

Mediator complex subunit 19 (Med19) binds to gene-specific regulatory factors and provides support for the basal RNA polymerase II transcription machinery. Med19 has been implicated in the progress of several cancers, and knockdown of its expression may inhibit the progression of these cancers [5–8]. However, the roles of Med19 on PCa growth and invasion are still obscure.

In the present study, we performed immunohistochemical (IHC) staining to compare the Med19 expression levels in human PCa tissues and adjacent benign prostate tissues. Then we knocked down the Med19 expression in PCa cell lines LNCaP and PC3 by using lentivirus siRNA to study the *in vitro* proliferation, cell cycle, anchor-independent growth, migration, and invasion of PCa cells. For the *in vivo* study, we inoculated the resultant PC3 cells subcutaneously to athymic nude mice to determine the tumor growth. For the mechanism, we performed quantitative reverse transcription polymerase chain reaction (Q-PCR) and Western blot assays to detect multiple genes relevant to proliferation, cell cycle, and metastasis.

## Materials and Methods

### IHC Staining

We collected 10 pairs of paraffin-embedded human PCa tissues and adjacent non-cancerous tissues (from 01-February-2014 to 30-August-2015) from Paraffin-embedded Tissue Bank of the Affiliated Yantai Yuhuangding Hospital of Qingdao University. The use of human tissues was approved by the Ethics Committee of the Affiliated Yantai Yuhuangding Hospital of Qingdao University. For IHC staining, after routine rehydration, antigen retrieval, and blocking, the sections were incubated in the primary antibody at 4°C overnight. The primary anti-Med19 antibody (1:200, Rabbit Polyclonal, HPA039912 Sigma-Aldrich) was recognized by the biotinylated secondary antibody and visualized by Vectastain avidin-biotin complex peroxidase system (ABC kit, VECTOR Laboratories) and peroxidase substrate 3,3'-diaminobenzidine kit (DAB kit, VECTOR Laboratories).

### Cell Culture and Lentivirus Infection

The human PCa cell lines LNCaP and PC3 were kind gifts from Dr. Shujie Xia of Shanghai Jiaotong University. All cells were cultured in RPMI 1640 supplemented with 10% fetal bovine serum (FBS), 100 units/ml penicillin, and 100 μg/mL streptomycin at 37°C in a humidified incubator containing 5% CO2.

To knock down the Med19 in LNCaP and PC3 cells, we performed the lentivirus infection strategy. Lentivirus containing siRNA targeting Med19 gene (5’-GTAGCTCTTTCAATCCTAT-3’) or nonsilencing control (5’-TTCTCCGAACGTGTCACGT-3’) were constructed by GeneChem, Shanghai, China. The day before infection, cells were plated at a density of 20~30% in a good condition. The above lentivirus (containing fluorescence) was treated to the LNCaP and PC3 cells according to the manufacture’s protocol. The culture medium was changed to normal medium 10 hours after infection. Three days later, the cells were subjected to fluorescence activated cell sorting (FACS) with flow cytometer for further experiments.

### Q-PCR

Total RNA was extracted and purified using Trizol (Takara, Carlsbad, CA). Three μg RNA was subjected to reverse transcription using Superscript III (TransGene, Beijing, China). Amplification was performed using SYBR green as fluorescent with the following PCR amplification conditions: 1 cycle at 95°C for 10 min, 45 cycles at 95°C for 15 sec and 60°C for 60 sec [9]. The relative expression of mRNA was calculated using the 2^-ΔΔCT^ to compare the expression levels among different samples. The primer sequences were as follows (5’-3’): Med19: Forward “TGCCAGGGATGATTGATCTG”, Reverse “TCTTCTTGGGAGGCTGAATATG”; IGF1R: Forward “CTGGCTCCGGAGGAGGGTCC”, Reverse “AATACATCTCCAGCCTCCTTAG”; N-Cadherin: Forward “TCAGGCGTCTGTAGAGGCTT”, Reverse “ATGCACATCCTTCGATAAGACTG”; E-cadherin: Forward “CTCCCAATACATCTCCCTTCAC”, Reverse “AGGTGGTCACTTGGTCTTTATT”; Vimentin: Forward “AGTCCACTGAGTACCGGAGAC”, Reverse “CATTTCACGCATCTGGCGTTC”; ZEB-2: Forward “CAAGAGGCGCAAACAAGCC”, Reverse “GGTTGGCAATACCGTCATCC”; Snail-1: Forward “TCGGAAGCCTAACTACAGCGA”, Reverse “AGATGAGCATTGGCAGCGAG”; Snail-2: Forward “CGAACTGGACACACATACAGTG”, Reverse “CTGAGGATCTCTGGTTGTGGT”; β-actin: Forward “GGCGGCACCACCATGTACCCT”, Reverse “AGGGGCCGGACTCGTCATACT”.

### Western Blot Analysis

The LNCaP-Med19-si/sc and PC3-Med19-si/sc cells were cultured in 10% FBS to 90% confluence, then were lysed in RIPA lysis buffer, separated on 8% SDS PAGE gel, and transferred to polyvinylidene difluoride (PVDF) membrane [9]. After blocked by 5% nonfat milk in PBST buffer, the membrane was incubated in the primary antibody and then in HRP-conjugated secondary antibodies. The primary antibodies used were: anti-Med19 (1:400, Rabbit Polyclonal, HPA039912 Sigma-Aldrich), anti-P27 (1:800, Rabbit Polyclonal, #2252 Cell Signaling Technology), anti-pAKT (1:1000, Rabbit Polyclonal, #9271 Cell Signaling Technology), anti-pPI3K (1:200, Rabbit Polyclonal, sc-293115 Santa Cruz Biotechnology), anti-GAPDH (1:500, Mouse monoclonal, sc-47724 Santa Cruz Biotechnology). Immunoreactive bands were detected by the chemiluminescent detection system (ECL, Pierce, Rockford, IL, USA)

### Methyl Thiazolyl Tetrazolium (MTT) Assay

The LNCaP-Med19-si/sc and PC3-Med19-si/sc cells were seeded in 24-well plates (1×10^4^ cells per well) and incubated with RPMI 1640 with 10% FBS in 37°C and 5% CO2 incubator with medium changed every 48 hours. The plates were stained with MTT (Sigma) at day 0, 2, 4, and 6 for 3 hours. The absorbency was read at a test wave length of 570 nm.

### Cell Cycle Analysis

Cells were fixed in 70% ice-cold ethanol and stained with propidium iodide (PI) (40 μg/ml) in RNase (100 μg/ml). Cells were analyzed using a FACScan flow cytometer (Becton Dickinson, San Jose, CA) and all groups were performed in triplet and statistically analyzed.

### Soft Agar Assay (Anchor-independent Colony Formation Aassay)

Each well of the six-well plate was laid with a base layer of 0.5 mL of 1% DNA grade agarose. The 1% melted DNA grade agar (at 40°C), 3×RPMI 1640 medium, and cell suspension were mixed by a ratio of 1:1:1, and come to a final cell concentration of 10^4^ cells/ml for PC3-Med19-si/sc cells and 5×10^4^ cells/ml for LNCaP-Med19-si/sc cells. One ml of the mixture was added on the top of the base agar. After coagulation, 2 ml RPMI 1640 supplemented with 10% FBS was added, and medium was replaced every 3 days to supply the nutrition. After 2 weeks’ culture in 37°C incubator, the colonies were stained by 1mg/ml INT (Sigma) solution.

### Invasion Assay

LNCaP-Med19-si/sc (1×10^5^) or PC3-Med19-si/sc (5×10^4^) cells in serum free RPMI 1640 medium was added to the cell culture inserts with microporous (8μm) membrane with or without Matrigel coating (control insert). RPMI 1640 medium containing 10% FBS was added to the bottom chamber. The cells were then incubated for 22 hours at 37°C, and the upper chamber was removed. The cells on the bottom of the upper chambers were stained with 1% toluidine blue, and the number of cells was counted under a microscope. The invasion ratio was the ratio of the number of cells invading through Matrigel coated insert membrane against the number of those migrated through non-coated insert membrane.

### Wound Healing Assay

The LNCaP-Med19-si/sc and PC3-Med19-si/sc cells were seeded in a 6-cm dish and cultured to confluency. The cell monolayer was scraped firmly with a plastic pipette tip and then left growing at 37°C. The "wounded" areas were photographed by a phase contrast microscopy in 0 and 24 hours after scraping. The percentage of wound healing area was calculated.

### Xenograft Model of Tumor Growth *in vivo*

Male immune-deficient BALB/c nude mice (4–6 weeks old) were purchased from Beijing Wei-tong Li-hua Laboratory Animals and Technology Ltd. The cells (1×10^6^) were suspended in 50μl Matrigel (BD Biosciences, Franklin Lakes, NJ, USA) and then implanted subcutaneously into the left flank (PC3-Med19-sc) and right flank (PC3-Med19-si) of nude mice. The inoculation sites were checked twice a week until tumors were palpable. Then the tumors were measured 3 times a week. All mice were observed daily for food, water, bedding, and general health conditions. The criteria of humane endpoints were as follows: Body Condition Score<2; any single tumor >2000 mm^3^, or multiple tumors >3000 mm^3^, or any tumor interferes with basic bodily functions; the tumor becomes ulcerated or necrotic; the animal’s ability to eat, drink, or breath is significantly impaired. All 6 mice survived well at 6 weeks after inoculation and were sacrificed by CO_2_. The tumors were harvested and weighed to compare between groups. The care and use of nude mice in this study were approved by the Ethics Committee of the Affiliated Yantai Yuhuangding Hospital of Qingdao University. All procedures abide by the 1964 Helsinki declaration and its later amendments or comparable ethical standards.

### Apoptosis Assay

Apoptotic cells were detected by terminal deoxynucleotidyl transferase-mediated biotinylated deoxyuridine triphosphate nickel end labeling (TUNEL) assay as recommended by the manufacturer (Roche). Briefly, paraffin-embedded tissue was deparaffinized, digested with proteinase K (20 μg/ml) for 15 minutes at room temperature, then was labeled for 60 minutes at room temperature and mounted (with DAPI).

### Statistical Analysis

Numerical data were presented as mean ± standard deviation. Statistical analysis between groups was performed by using two-sided Student’s T test. *P* values <0.05 were considered statistically significant.

## Results

### Med19 Expression Level Was Elevated in PCa Tissues

**Fig 1:**
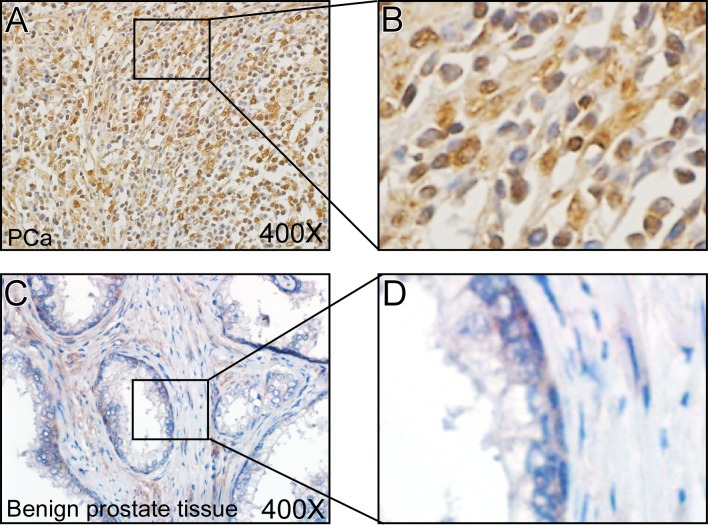
Med19 expression level was elevated in PCa tissues. Med19 IHC staining was performed in 10 pairs of PCa and adjacent benign prostate tissues. The Med19 expression level was much higher in PCa tissues (A, B) than in adjacent benign tissues (C, D). (A) and (C) were taken at 400×magnification, (B) and (D) were local amplified pictures.

We performed IHC staining to test the Med19 expression in 10 pairs of PCa tissues and adjacent benign tissues, and found that Med19 expression level was much higher in PCa tissues (Fig 1A and 1B) than in adjacent benign tissues (Fig 1C and 1D), which indicated that Med19 was up-regulated in PCa tissues. This data encouraged us to continue studying the Med19 roles by using the PCa cell lines LNCaP and PC3.

### Knockdown of Med19 with Lentiviral Infection in PCa Cells

**Fig 2:**
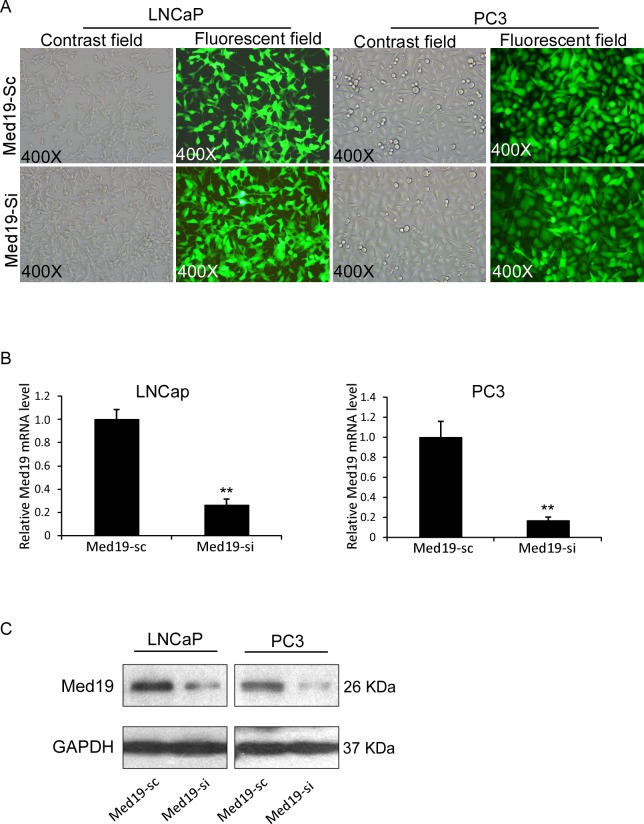
Establishment and characterization of LNCaP-Med19-si/sc and PC3-Med19-si/sc cells. (A) After lentivirus infection and FACS sorting, the transduction efficiency was confirmed by determining the GFP expression in a fluorescent microscope. The GFP positive rate was more than 95% in all cells. (B) In Q-PCR assay, the relative Med19 expression levels were 26.18±5.27% in LNCaP-Med19-si cells (n = 3, **P<0.01), and 16.88±3.40% in PC3-Med19-si cells (n = 3, **P<0.01). (C) In Western blot assay, the Med19 expression level was dramatically decreased in LNCaP-Med19-si and PC3-Med19-si cells. GAPDH was used as an internal control.

Since Med19 expresses at high levels in both LNCaP and PC3 cells, we performed lentivirus siRNA knockdown strategy. The LNCaP and PC3 cells were infected with lentivirus that contained either siMed19 or control scramble RNA, then were sorted by FACS to establish relatively stable cells of LNCaP-Med19-si and PC3-Med19-si, as well as their control cells of LNCaP-Med19-sc and PC3-Med19-sc. Efficient transduction was confirmed by determining the GFP expression in a fluorescent microscope (Fig 2A). The GFP positive rate was more than 95% in both cell lines. In mRNA level, the relative Med19 expression was 26.18±5.27% in LNCaP-Med19-si cells, and 16.88±3.40% in PC3-Med19-si cells (Fig 2B). We further determined the Med19 expression level with Western blot. Compared to the control cells, the Med19 protein expression level was dramatically decreased in LNCaP-Med19-si and PC3-Med19-si cells (Fig 2C).

### Knockdown of Med19 Inhibited PCa Cells Growth via Cell Cycle Arrestment

**Fig 3:**
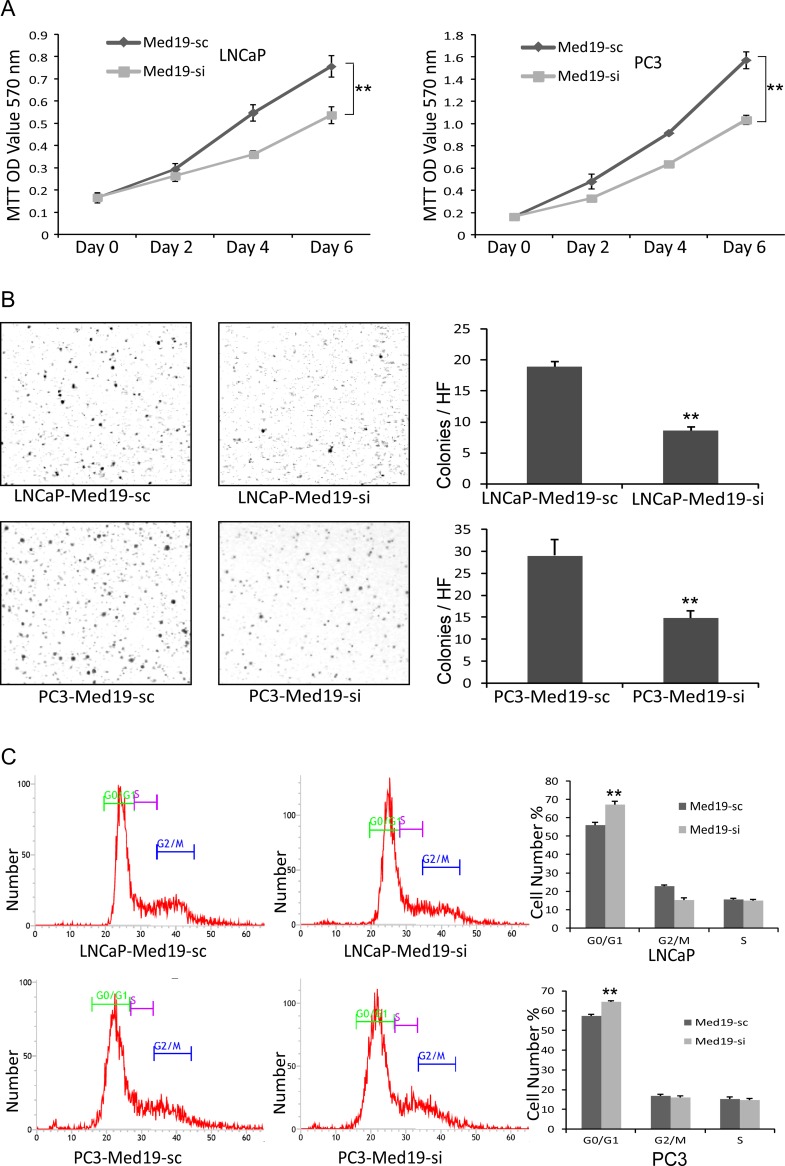
Knockdown of Med19 inhibited cell growth via arresting cell cycle. (A) In MTT assay, the growth curves showed the LNCaP-Med19-si and PC3-Med19-si cells grew much slower than the control cells. (n = 3, **P<0.01). (B) In colony formation assay (soft agar assay), the LNCaP-Med19-si cells formed 8.61±0.51 colonies/HF, and the LNCaP-Med19-sc cells formed 18.89±0.82 colonies/HF. (n = 3, **P<0.01). The PC3-Med19-si cells formed 14.89±1.51 colonies/HF, and the PC3-Med19-sc cells formed 29.06±3.62 colonies/HF. (n = 3, **P<0.01). (C) In flow cytometry cell cycle analysis, the proportion of G0/G1 phase cells was significantly increased in LNCaP-Med19-si and PC3-Med19-si cells. The proportion of G0/G1 was 67.29±1.70% in LNCaP-Med19-si cells and 56.23±1.46% in LNCaP-Med19-sc cells (n = 3, **P<0.01). The proportion of G0/G1 was 64.39±0.62% in PC3-Med19-si cells and 57.29±0.97% in PC3-Med19-sc cells (n = 3, **P<0.01).

In MTT assay, the LNCaP-Med19-si and PC3-Med19-si cells grew much slower than their control cells (Fig 3A). MTT assay could only reflect the cell growth ability in attached conditions. For tumor cells, the anchor-independent growth is an important property for their malignancy. The soft agar assay was performed to evaluate the anchor-independent growth of the PCa cells. After 2 weeks culture in soft agar, the LNCaP-Med19-si and PC3-Med19-si cells formed much less colonies than control cells (Fig 3B). To determine whether down-regulation of Med19 could impact the cell cycle of PCa cells, the flow cytometry cell cycle analysis was performed. Compared with control cells, the proportion of G0/G1 phase cells was significantly increased in the LNCaP-Med19-si and PC3-Med19-si cells (Fig 3C), which indicated that knockdown of Med19 resulted in cell cycle arrest in G0/G1 phase.

### Knockdown of Med19 Inhibited PCa Cell Invasion and Migration *in vitro*

**Fig 4:**
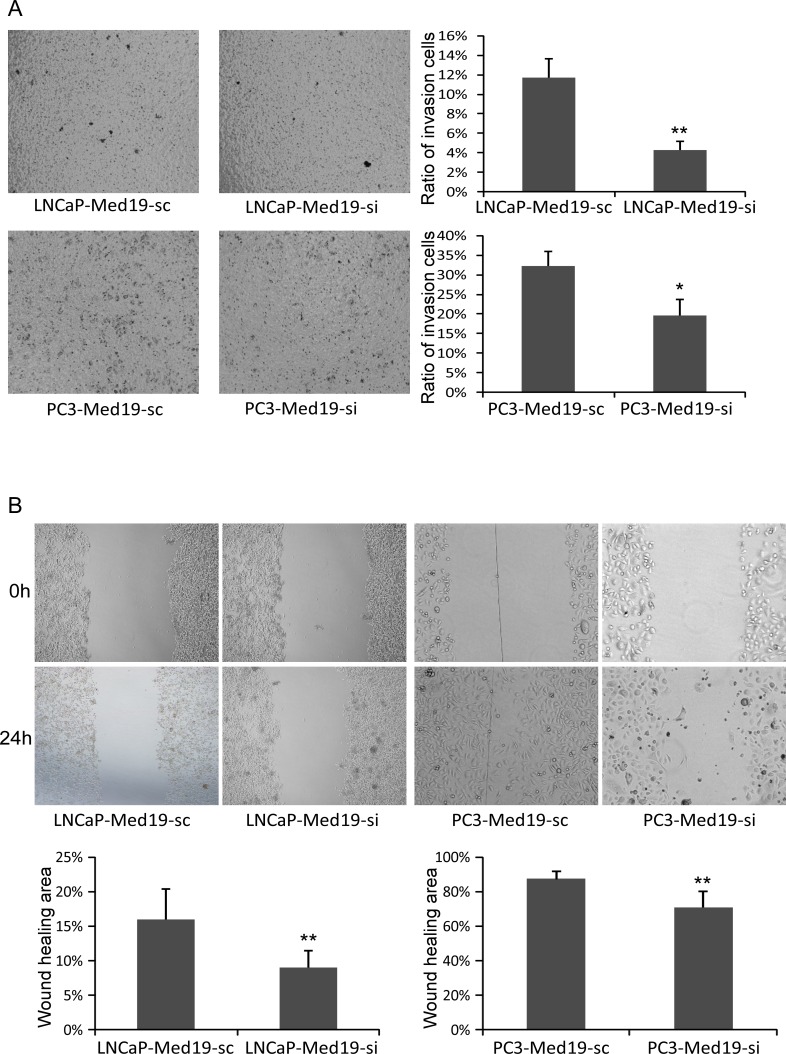
Knockdown of Med19 inhibited PCa cells invasion and migration *in vitro*. (A) In invasion assay, the invasion ratio was 4.26±0.92% in LNCaP-Med19-si cells and 11.74±1.94% in LNCaP-Med19-sc cells (n = 3, **P<0.01). The invasion ratio was 19.71±4.00% in PC3-Med19-si cells and 32.27±3.72% in PC3-Med19-sc cells (n = 3, *P<0.05). (B) In “wound healing” migration assay, after 24 hours culture, the percentage of wound healing area was 8.83±2.56% in LNCaP-Med19-si cells and 15.83±4.62% in LNCaP-Med19-sc cells (n = 3, **P<0.01), 70.83±9.37% in PC3-Med19-si cells and 87.33±4.63% in PC3-Med19-sc cells (n = 3, **P<0.01).

Invasion and migration abilities are important for the metastasis of tumor cells. An intro chamber invasion assay was performed to detect the invasion ability of PCa cells. After 22 hours’ culture in the Boyden chambers, much fewer LNCaP-Med19-si cells (invasion ratio of 4.26±0.92% v.s.11.74±1.94% *P*<0.01) and PC3-Med19-si cells (invasion ratio of 19.71±4.00% v.s.32.27±3.72%, *P*<0.05) invaded through the Matrigel membrane than their control cells (Fig 4A). To test the migration ability, a wound healing assay was performed. The PCa cells in culture dishes were scraped with a plastic pipette tip to generate the same size of “wound” area, then continue to culture for 24 hours. Compared to control cells, the wound healing areas were much smaller in the LNCaP-Med19-si cells (8.83±2.56% v.s.15.83±4.62%, *P*<0.01) and PC3-Med19-si cells (70.83±9.37% v.s.87.33±4.63%, *P*<0.01) (Fig 4B).

### Knockdown of Med19 Inhibited PCa Progression *in vivo*

**Fig 5:**
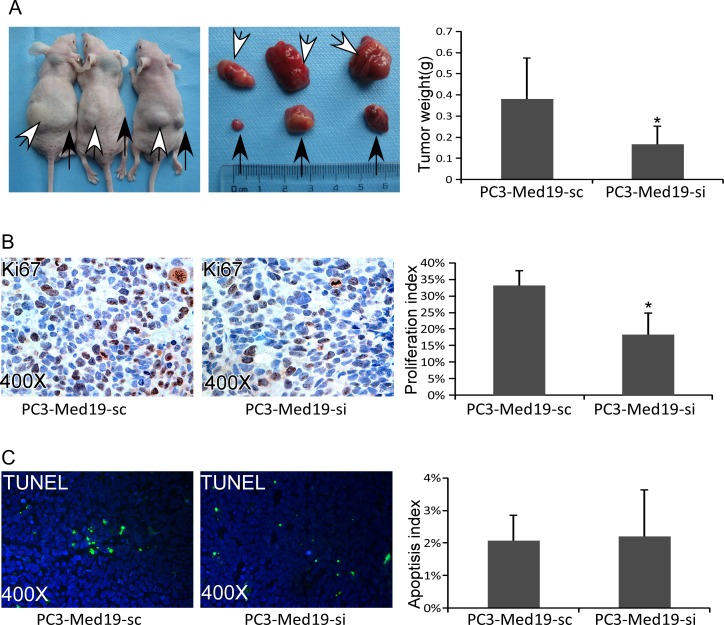
Knockdown of Med19 inhibited PCa progression in nude mice. (A) Six weeks after inoculation, PC3-Med19-si (black arrows showed tumors in the right flank) and PC3-Med19-sc (white arrows showed tumors in the left flank) formed tumors in nude mice. The weight of PC3-Med19-si and PC3-Med19-sc tumors was 0.17±0.08g v.s. 0.38±0.19g (n = 6, *P<0.05). (B) In Ki67 IHC staining, the PC3-Med19-si tumors had fewer positive cells than PC3-Med19-sc tumors. The proliferation index was 18.30±6.55% in PC3-Med19-si tumors and 33.22±4.36% in control tumors (n = 6, *P<0.05). (C) In TUNEL assay, the apoptotic cells were similar in both tumors. The apoptosis index in PC3-Med19-si and PC3-Med19-sc tumors was 2.21±1.43% v.s. 2.08±0.78% (n = 6, *P*>0.1).

To confirm the Med19 roles in PCa tumor growth *in vivo*, a xenograft tumor model was established in nude mice. The PCa cells were inoculated subcutaneously into nude mice flank areas. Since the LNCaP cells were difficult to form tumor in nude mice, we only collected the data of PC3 cells. Six weeks after cells inoculation, the tumors were harvested and weighed. The PC3-Med19-si cells formed much smaller tumors than their control tumors (0.17±0.08g v.s.0.38±0.19g, *P*<0.05) (Fig 5A). To test the proliferation rate of the xenograft tumors, Ki67 IHC staining was performed. There were fewer Ki67 positive cells in PC3-Med19-si tumors than control tumors. The proliferation index was 18.30±6.55% in PC3-Med19-si tumors, but 33.22±4.36% in control tumors, *P*<0.05 (Fig 5B). The TUNEL assay was used to further determine the apoptotic cells in the xenograft tumors. However, the ratio of apoptotic cells did not come to a statistical significance between groups (Fig 5C).

### Knockdown of Med19 Altered the Cell Growth, Cell Cycle, and Epithelial–Mesenchymal Transition (EMT) Related Genes

**Fig 6:**
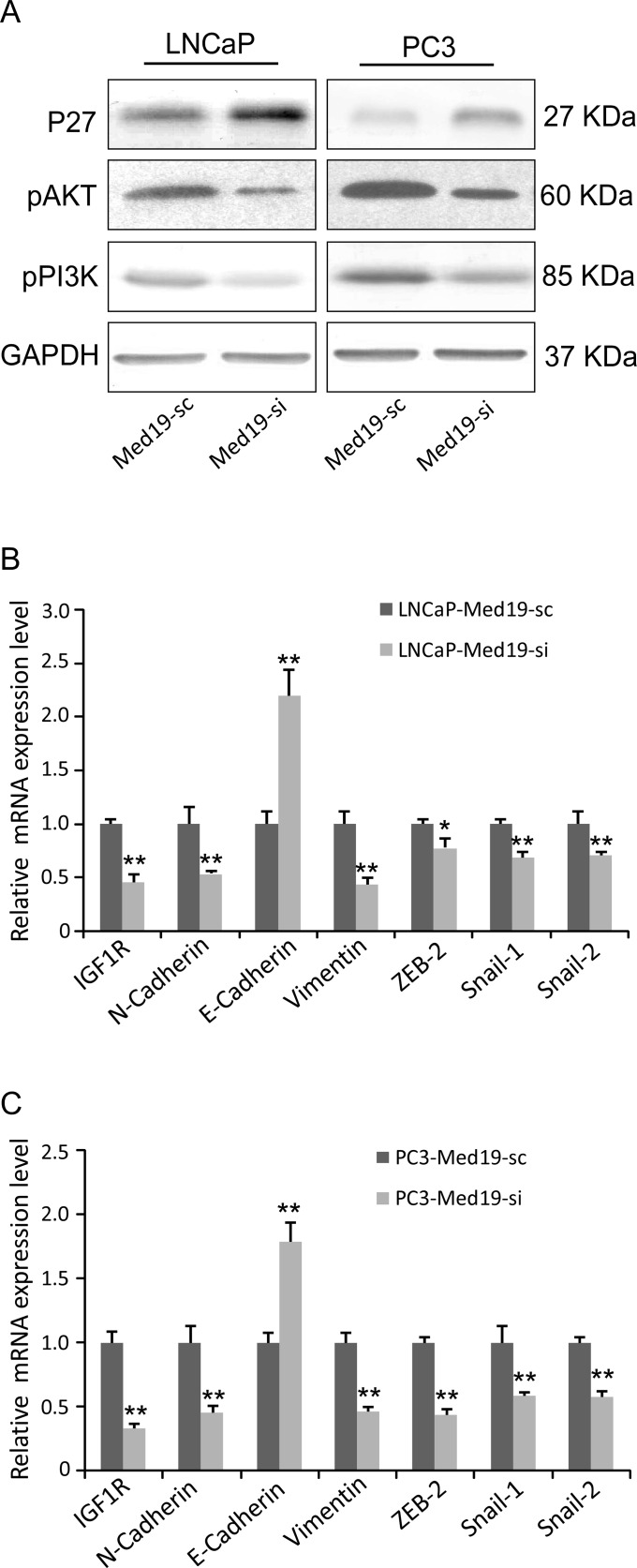
Knockdown of Med19 altered multiple genes expression in PCa cells. (A) In Western blot assay, the pAKT and pPI3K expression levels were lower, and the P27 expression level was higher in the Med19 knockdown cells. GAPDH was used as an internal control. (B, C) In Q-PCR assay, the IGF1R, N-Cadherin, Vimentin, ZEB2, Snail-1, and Snail-2 were down-regulated, and the E-Cadherin was up-regulated in both LNCaP-Med19-si and PC3-Med19-si cells (n = 3, *P<0.05, **P<0.01).

In Western blot assay, the pAKT and pPI3K expression levels decreased, and P27 expression level increased in the Med19 knockdown cells (Fig 6A). In Q-PCR analysis, the IGF1R expression was down-regulated (Fig 6B and 6C), and many EMT related genes including E-Cadherin, N-Cadherin, Vimentin, ZEB2, Snail-1, and Snail-2 were altered in the Med19 knockdown cells (Fig 6B and 6C).

## Discussion

Med19 is a component of the Mediator complex, which involved in the transcription regulation of nearly all RNA polymerase II-dependent genes [10]. Med19 is important in stabilizing the entire Mediator complex, making it critical to the transcriptional regulation [11]. Absence of Med19 may decrease the binding affinity of Mediator with RNA Polymerase II [11].

Med19 has been reported in the progress of several cancers, and knockdown of its expression may inhibit the growth and spread of these cancers. In bladder urothelial carcinoma, the Med19 was reported to promote cancer cell growth, bone metastasis, and invasiveness by regulating cell cycles and bone morphogenetic protein 2 [8, 12]. In breast cancer, Med19 may promote cell proliferation by regulating CBFA2T3/HEB expression [13]. In lung cancer, MED19 could promote tumor proliferation, tumorigenesis, metastasis, and enhance chemo-sensitivity to cisplatin in non-small cell lung cancer cells [14–16]. The Med19 has been reported to promote tumor growth and/or metastasis in ovarian cancer, gastric cancer, pancreatic cancer, osteosarcoma, and liver cancer [5–7, 17–18]. However, there are few reports about the roles of Med19 in PCa. Cui et al [19] reported Med19 could promote the proliferation and tumorigenesis and induce apoptosis in human prostate cancer cells. Imberg-Kazdan et al [20] found that depletion of HIPK2 and MED19 in human prostate cancer cells decreased AR target gene expression and reduced the proliferation of androgen-dependent and castration-resistant prostate cancer cells.

In the present study, we performed Me19 IHC staining on human PCa tissues and adjacent benign prostate tissues, and found Med19 expression level was elevated in PCa tissues. We then knocked down the Med19 expression in PCa cell lines LNCaP and PC3 by using lentivirus siRNA. The cell proliferation, anchor-independent growth, migration, invasion abilities were suppressed in Med19 knockdown PCa cells. More Med19 knockdown PCa cells were arrested in G0/G1 phase. By using nude mice xenograft PCa model, we found the Med19 knockdown PCa cells formed smaller tumors and had lower proliferation index. For the mechanism, we found Med19 could regulate the expression of multiple genes relevant to tumor growth and metastasis, including P27, pAKT, pPI3K, IGF1R, E-Cadherin, N-Cadherin, Vimentin, ZEB2, Snail-1 and Snail-2.

PI3K/AKT is important in regulating the cell cycle [21]. PI3K activation phosphorylates and activates AKT, and AKT has a number of downstream effects including inhibition of p27 [22]. P27, also known as Kip1, is a cyclin-dependent kinase inhibitor [23]. It is well known that P27 could suppress tumor cells proliferation by regulating the cell cycle [24]. IGF1R, belonging to the large class of tyrosine kinase receptors, plays promotional roles in PCa progression [25]. Our date suggested that the Me19 could promote PCa tumor growth by regulating pAKT, pPI3K, P27, and IGF1R expression level.

In the EMT process, epithelial cells lose their polarity and gain migratory and invasive properties. Initiation of cancer metastasis requires invasion, which is enabled by EMT [26]. In our study, the EMT related genes N-Cadherin, Vimentin, ZEB2, Snail-1 and Snail-2 expression levels decreased and E-Cadherin expression level increased in Med19 knockdown cells. This suggested that Me19 could increase PCa tumor metastatic abilities by promoting EMT.

Overall, in the present study, we found that Med19 could promote PCa growth and metastasis by regulating the cell proliferation, cell cycle, and EMT-related genes. Targeting the Med19 in PCa cells could inhibit the PCa growth and metastasis, and might be a therapeutic option for PCa in the future.

## Supporting Information

S1 Fig The volume of subcutaneous tumors in nude mice. Six weeks after inoculation, PC3-Med19-si and PC3-Med19-sc formed tumors in nude mice. The PC3-Med19-si tumor volume was 211.8±123.7mm^3^, and the PC3-Med19-sc tumor volume was 462.2±233.3mm^3^ (n = 6, *P<0.05). The tumor volume was calculated by the formula “Volume = π/6 (L×W×H)”.
(TIF)

S2 Fig The expression of apoptosis related genes was not altered in Med19 knockdown cells. In Q-PCR assay, the Bax, Bcl-2, Caspase-3, and Caspase-8 expression levels were not altered in LNCaP-Med19-si and PC3-Med19-si cells (n = 3, *P<0.05). The primer sequences are as follows: Bax: Forward “TCATGGGCTGGACATTGGAC”, Reverse “GAGACAGGGACATCAGTCGC”; Bcl-2: Forward “TGGATGACTGAGTACCTGAACC”, Reverse “CGCATCTCGGACCTGTGG”; Caspase-3: Forward “GCGGTTGTAGAAGTTAATAAAGGT”, Reverse “ATTCGCTTCCATGTATGATCTTTGG”; Caspase-8: Forward “GGAACTTCAGACACCAGGCA”, Reverse “CCTCCGCCAGAAAGGTACAG”.
(TIF)
