# Supplementary figures and images for: Knockdown of Mediator Complex Subunit 19 Suppresses the Growth and Invasion of Prostate Cancer Cells
Source: PLoS One. 2017 Jan 26;12(1):e0171134. doi: 10.1371/journal.pone.0171134 (PMC5270333; doi:10.1371/journal.pone.0171134)

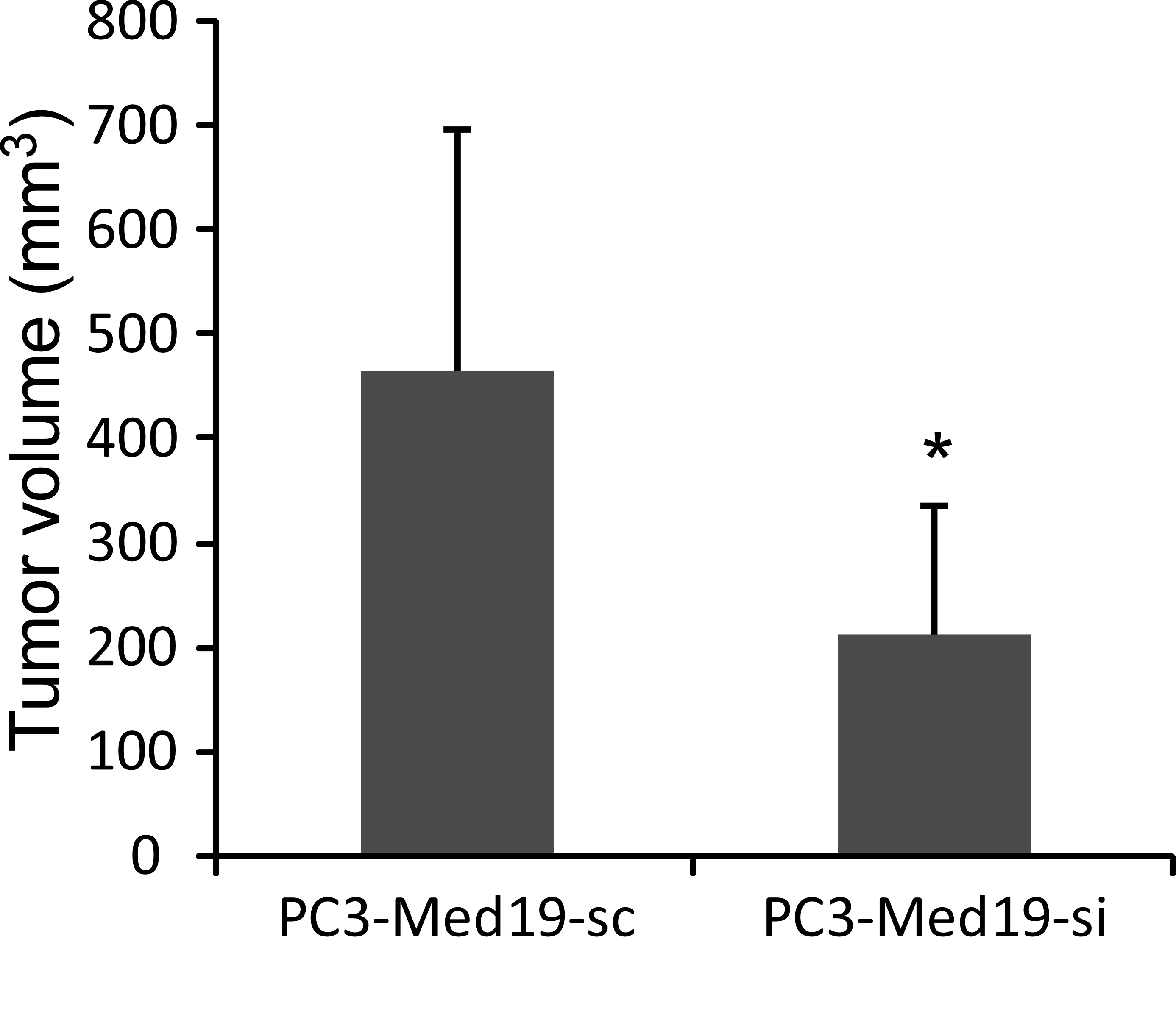

Supplement: S1 Fig — Six weeks after inoculation, PC3-Med19-si and PC3-Med19-sc formed tumors in nude mice. The PC3-Med19-si tumor volume was 211.8±123.7mm3, and the PC3-Med19-sc tumor volume was 462.2±233.3mm3 (n = 6, *P<0.05). The tumor volume was calculated by the formula “Volume = π/6 (L×W×H)”. (TIF) [file pone.0171134.s001.tif]

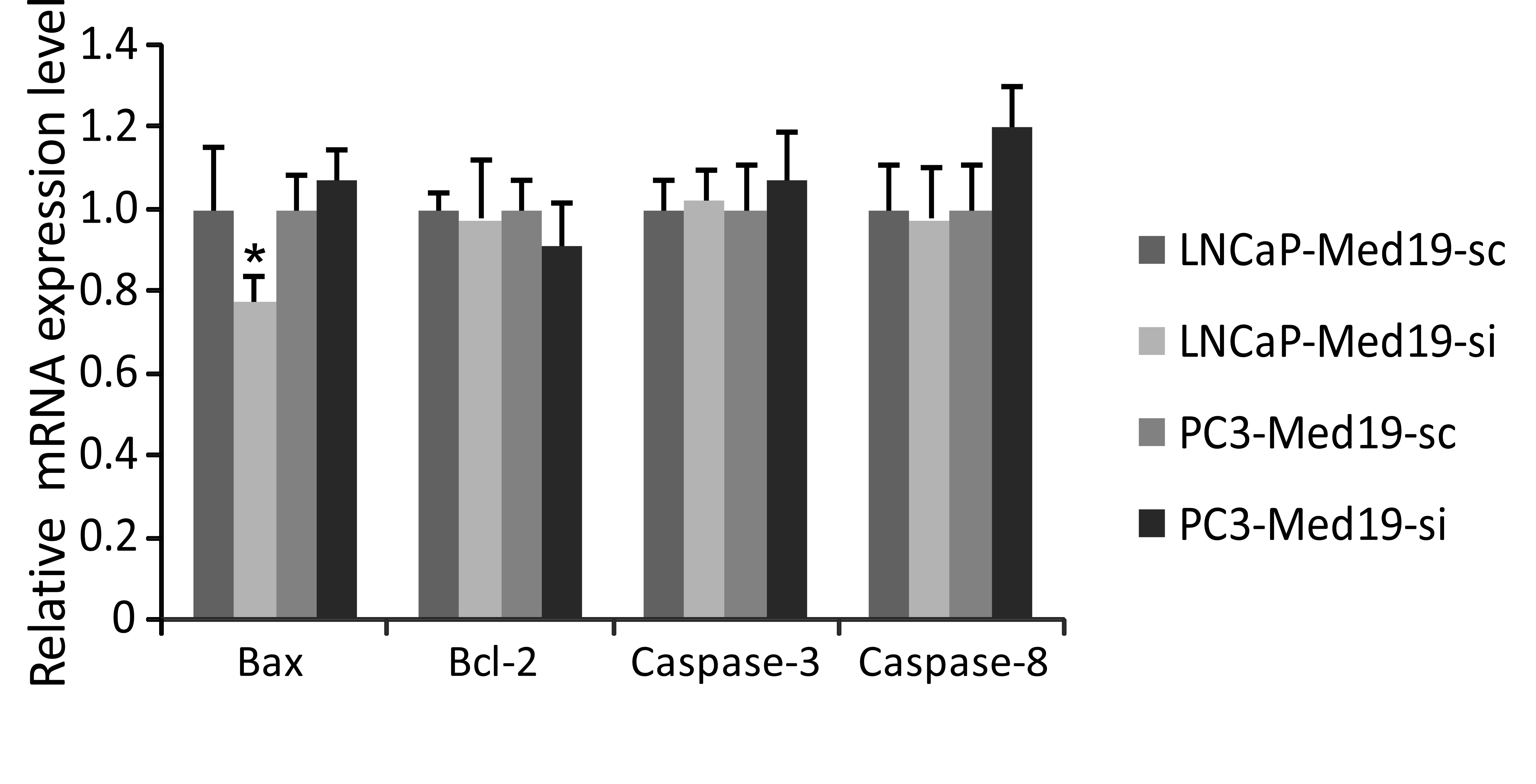

Supplement: S2 Fig — In Q-PCR assay, the Bax, Bcl-2, Caspase-3, and Caspase-8 expression levels were not altered in LNCaP-Med19-si and PC3-Med19-si cells (n = 3, *P<0.05). The primer sequences are as follows: Bax: Forward “TCATGGGCTGGACATTGGAC”, Reverse “GAGACAGGGACATCAGTCGC”; Bcl-2: Forward “TGGATGACTGAGTACCTGAACC”, Reverse “CGCATCTCGGACCTGTGG”; Caspase-3: Forward “GCGGTTGTAGAAGTTAATAAAGGT”, Reverse “ATTCGCTTCCATGTATGATCTTTGG”; Caspase-8: Forward “GGAACTTCAGACACCAGGCA”, Reverse “CCTCCGCCAGAAAGGTACAG”. (TIF) [file pone.0171134.s002.tif]
